# Reduction of Severe Acute Maternal Morbidity and Maternal Mortality in Thyolo District, Malawi: The Impact of Obstetric Audit

**DOI:** 10.1371/journal.pone.0020776

**Published:** 2011-06-03

**Authors:** Thomas van den Akker, Jair van Rhenen, Beatrice Mwagomba, Kinke Lommerse, Steady Vinkhumbo, Jos van Roosmalen

**Affiliations:** 1 Thyolo District Health Office, Ministry of Health, Thyolo, Malawi; 2 Department of Medical Humanities, EMGO Institute for Health and Care Research, VU University Medical Center, Amsterdam, The Netherlands; 3 Department of Obstetrics, Leiden University Medical Center, Leiden, The Netherlands; University of Oxford, United Kingdom

## Abstract

**Background:**

Critical incident audit and feedback are recommended interventions to improve the quality of obstetric care. To evaluate the effect of audit at district level in Thyolo, Malawi, we assessed the incidence of facility-based severe maternal complications (severe acute maternal morbidity (SAMM) and maternal mortality) during two years of audit and feedback.

**Methodology/Principal Findings:**

Between September 2007 and September 2009, we included all cases of maternal mortality and SAMM that occurred in Thyolo District Hospital, the main referral facility in the area, using validated disease-specific criteria. During two- to three-weekly audit sessions, health workers and managers identified substandard care factors. Resulting recommendations were implemented and followed up. Feedback was given during subsequent sessions. A linear regression analysis was performed on facility-based severe maternal complications. During the two-year study period, 386 women were included: 46 died and 340 sustained SAMM, giving a case fatality rate of 11.9%. Forty-five cases out of the 386 inclusions were audited in plenary with hospital staff. There was a reduction of 3.1 women with severe maternal complications per 1000 deliveries in the district health facilities, from 13.5 per 1000 deliveries in the beginning to 10.4 per 1000 deliveries at the end of the study period. The incidence of uterine rupture and major obstetric hemorrhage reduced considerably (from 3.5 to 0.2 and from 5.9 to 2.6 per 1000 facility deliveries respectively).

**Conclusions:**

Our findings indicate that audit and feedback have the potential to reduce serious maternal complications including maternal mortality. Complications like major hemorrhage and uterine rupture that require relatively straightforward intrapartum emergency management are easier to reduce than those which require uptake of improved antenatal care (eclampsia) or timely intravenous medication or HIV-treatment (peripartum infections).

## Introduction

Despite countless conferences and policy documents, an unacceptably high number of women does not survive childbirth in many low-income countries, particularly in sub-Saharan Africa [1]. Women die at home because of delay in the decision to seek care (‘phase-I delay’), or during transport to the appropriate level of health facility (‘phase-II delay’). Moreover, even if women make it to the appropriate level of care in time, the often considerable time elapse until onset of correct treatment (‘phase-III delay’) leads to mortality or serious morbidity [2].

In order to improve obstetric care and reduce delay, critical incident audit and feedback have been recommended by the World Health Organization (WHO) and other institutions [3], [4]. Audit can be defined as any summary of clinical performance of health care over a specified period of time, and is commonly used to identify substandard care factors including modifiable behaviors of health professionals [4]. Combined with effective feedback to these professionals and other stakeholders, audit is suggested as a potentially useful strategy to improve health care delivery [4], [5].

Maternal and perinatal mortality reviews are currently applied in many institutions around the globe [4]. Audit of severe acute maternal morbidity (SAMM) is increasingly perceived as a useful complementary [6]. However, despite their widespread institutionalization, empirical evidence for benefits of audit and feedback remains limited [4], [5].

Especially in low-resource countries, which carry by far the highest burden of maternal mortality and morbidity, the effects of audit are poorly understood [4]. In Malawi, where the maternal mortality ratio stood at a devastating 1,140 per 100,000 live births in 2008 [1], only one observational study suggested that audit may have contributed to a modest reduction in facility-based maternal mortality in three districts over a three-year period between 2005 and 2007 [7].

In Thyolo District, Malawi, systematic audit and feedback started being performed in August 2007. Evaluation of one-year audit results showed a reduction in the incidence of uterine rupture at district hospital level by 68% (from 19.2 to 6.1 per 1000 deliveries; OR 0.32, 95% CI: 0.16–0.63) [8]. Following this positive outcome, we sought to examine whether this achievement had been sustained and quantify the effect of audit on overall district-wide facility-based maternal health outcomes over a longer period of time. This evaluation became even more important in light of a considerable increase in intrapartum care provided in district health facilities during the study period, putting the quality of care in these under-resourced sites to an additional test [9].

Therefore, we undertook this study to evaluate changes in incidence of facility-based maternal mortality and SAMM during a two-year period of intensive audit and feedback in the district.

## Materials and Methods

### Ethics Statement

The study was performed in full accordance with the guidelines for operational research of the National Research Council and the Health Sciences Research Committee of the Ministry of Health of Malawi and with the Helsinki declaration of 1975, as revised in 1983 [10], [11]. Verbal approval was obtained from the National Health Sciences Research Committee from the Ministry of Health, Malawi, which ruled that formal approval was not necessary for this type of study. In addition, the National Health Sciences Research Committee as well as the District Health Office of the Ministry of Health ruled that written consent was not necessary for this type of operational research which should in fact be routine practice in any Malawian district hospital in order to monitor clinical performance [7]. Nevertheless, verbal informed consent was obtained from all included women or their relatives (in case of maternal mortality) before collecting their information into the database. All results were de-identified and none of the information collected in the database could be traced back to any individual patient. The District Health Office of the Ministry of Health took part in the study design and ensured that the study was performed conform national guidelines.

### Study design

This was a facility-based prospective cohort study.

### Study period and setting

From September 1^st^, 2007, to September 1^st^, 2009, this study was performed in Thyolo District, a predominantly rural area in Southern Malawi, a very low income country in sub-Saharan Africa. The district had a population of 587 455 in 2008 and an HIV-prevalence rate of 21% in 2004 [12].

The publicly-funded Thyolo District Hospital is the largest health facility and most important secondary referral center in the area; 4363 deliveries were conducted in the facility in 2008. During the same year, an additional 11 556 health facility deliveries took place in 28 smaller government, mission and private facilities throughout the district, adding up to a total of 15 919 facility deliveries, almost 55% of the estimated 29 400 deliveries in the district that year (based on a birth rate of 50‰) [13]. Between 2007 and 2009 the number of women delivering at a health facility steadily increased, probably due to the provision of culturally-sensitive non-monetary incentives. The increase was largest in peripheral primary care centers compared to the semi-urban district hospital (94% versus 38% in two years). The increase in the district hospital could be attributed primarily to increased referrals from peripheral sites [9].

Due to a very low number of physicians in the district, clinical officers –a cadre of “non-physician clinicians”- perform the bulk of clinical work, including major surgery such as caesarean section [14]. With limited availability of registered nurse/midwives, their workload is high and most deliveries are conducted by lower nursing cadres. These clinical and nursing cadres with less professional training have expressed particular interest in continuous professional development at the workplace such as provided by audit and feedback sessions [15].

### Study participants

We included all cases of maternal mortality and SAMM that occurred in any of the district health facilities into the study which was labeled “4M-study”: maternal mortality and morbidity in Thyolo District. All mortality and morbidity cases were included regardless of additional maternal or gestational factors (e.g. underlying condition of the mother, gestational age at the time of the complication, multiple gestation etc.).

We used the definition of maternal mortality as given in the 10^th^ version of the International Classification of Diseases: the death of a woman while pregnant or within 42 days of termination of pregnancy, irrespective of the duration and site of the pregnancy, from any cause related to or aggravated by the pregnancy or its management but not from accidental or incidental causes [16].

At present, there is no universally accepted case definition of SAMM [17], [18]. In the literature, three types of inclusion criteria for SAMM are reported: (1) organ-system failure based criteria; (2) management-based criteria (e.g. emergency hysterectomy and/or admission into an intensive care unit); (3) disease-specific criteria [17], [19]–[23]. The WHO is in the process of comparing these three types of criteria for SAMM in order to arrive at a universally acceptable definition [24]. In Malawian district hospitals without intensive care units or extensive diagnostic capacity, disease-specific criteria are considered to be the most convenient and comprehensive [17].

Therefore, we chose disease-specific criteria and included pregnant and recently delivered women up to 6 weeks postpartum with one or more of the following entities:

1. uterine rupture, defined as the occurrence of clinical symptoms (pain, fetal distress, acute loss of contractions, hemorrhage) or intrauterine fetal death that led to laparotomy, at which the diagnosis was confirmed, or laparotomy for uterine rupture after vaginal birth [4]; to this definition we added rupture confirmed by autopsy or clinical symptoms with a high suspicion of rupture in case of death;
2. eclampsia *or* severe pre-eclampsia, defined as any case of pre-eclampsie with a maternal indication for termination of pregnancy;
3. major obstetric hemorrhage (including hemorrhage from complicated abortions and ectopic pregnancies) defined as a fulfilled need for transfusion of at least two units of 450 milliliters of whole blood (we adjusted the commonly cited criterion of four units because of scarcity of blood for transfusion in the local setting) *or* a hemoglobin level below 6 g/dl measured after vaginal bleeding *or* an estimated blood loss of more than 1 liter;
4. severe obstetric and non-obstetric peripartum infections, defined as all infections for which intravenous antibiotics or intravenous anti-malarials were prescribed or surgical treatment was performed, as well as neoplasms resulting primarily from HIV-infection (e.g. Kaposi's sarcoma and HIV-associated lymphoma);
5. any other complication the clinician considered severe, with the qualification ‘severe’ confirmed by at least two senior clinicians.

These criteria were modified for the local setting from similar international studies [21], [23]–[26].

### Data collection and analysis

Patients were identified and included into the study by clinicians and nurses who received assistance from medical students from Leiden University, the Netherlands, during daily ward rounds in the obstetric department and thrice-weekly rounds in the women's medical and surgical wards. Each inclusion was also verified with a senior clinician. Upon discharge or upon confirmation of death of any hospitalized patient, medical records (antenatal card, labor graph and medical case file) of each woman were again screened for relevant diagnoses according to SAMM criteria. Staff responsible for including women into the study were unaware of the specific objective to evaluate overall trends in maternal outcome.

We postulated that cases seen at the district hospital (the main referral institution for primary level facilities in the district) comprised the overwhelming majority of SAMM and maternal mortality cases occurring in district health facilities. According to district health protocols, all women with severe maternal complications should be referred to secondary level for appropriate observation and treatment. In order to justify our hypothesis and control for underreporting at primary care level, we searched records of ten smaller primary care centers for cases fitting inclusion criteria at the end of the study period. We were not able to identify any further inclusions from records in these facilities other than cases that had been referred to the district hospital and that had already been included into the study at that level.

All relevant data were entered into a Microsoft Excel database and, based on the assumption that audit and feedback would account for a decrease in maternal complications, linear regression analyses were performed with IBM® SPSS® Statistics 17.0 software package with “quarter” as independent variable, and maternal complications, maternal mortality and SAMM per 1000 deliveries per quarter as dependent variables. We also assessed trends for each individual SAMM diagnosis and for the number of HIV-positive women included into the study.

### Audit

Audit sessions were held every two to three weeks, involving members of each cadre of clinical staff, as well as members of the District Health Management Team. External gynecologist-obstetricians were occasionally invited to join for audit. During audit sessions, cases of maternal mortality and SAMM were presented by hospital staff on a rotational basis and discussed by all available health workers and managers, usually between 30 and 45 staff. The hospital team of clinicians and nurses who were taking part in the audit sessions and assessed clinical care remained almost unchanged throughout the two-year study period. Those responsible for supporting departments such as the pharmacy and the transport system joined from time to time. Intention was to evaluate all cases which senior clinicians or audit organizers considered to be of educational value. Occasionally, audits were also held at smaller peripheral health centers.

The benchmark against which substandard care was measured were national and district health office protocols, as well as WHO-protocols on all relevant conditions [27], [28]. If health workers identified substandard care factors, they were asked to recommend measures for improvement. Implementation of these recommendations, depending on the nature of each suggested measure, was performed by the management team as well as by individual professionals. Evaluation of these initiatives took place on a regular basis during subsequent audit sessions.

## Results

During the two-year study period, 386 women with serious pregnancy-related complications presented to the district hospital: 46 women died and 340 sustained SAMM, giving a case fatality rate of 11.9% (46 out of 386 cases) and a morbidity/mortality-ratio of 7.4.

**Figure 1:**
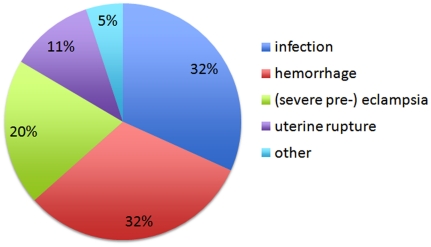
Diagnoses comprising SAMM; n = 375.

In the 340 women who sustained SAMM (101 (30%) of whom were HIV-positive), 375 SAMM diagnoses were made: 31 (8.3%) of these women had two inclusion diagnoses, and two women had three. These 375 SAMM diagnoses were comprised of: 119 (32%) severe infections or HIV-related complications, 119 (32%) cases of major obstetric hemorrhage, 75 (20%) cases of eclampsia or severe pre-eclampsia, 43 (11%) uterine ruptures, and 19 (5%) other severe maternal complications according to the clinician (Figure 1, Box S1).

Of the 119 women with infection-related morbidity (55 (46%) of whom were HIV-positive), 81 had an obstetric infection (36 (44%) HIV-positive), 37 a non-obstetric infection (18 (49%) HIV-positive) and one was diagnosed with HIV-related Kaposi 's sarcoma. Of the 119 cases of major obstetric hemorrhage, eight (7%) were caused by ectopic pregnancies and seven (6%) by complications of abortion.

**Figure 2:**
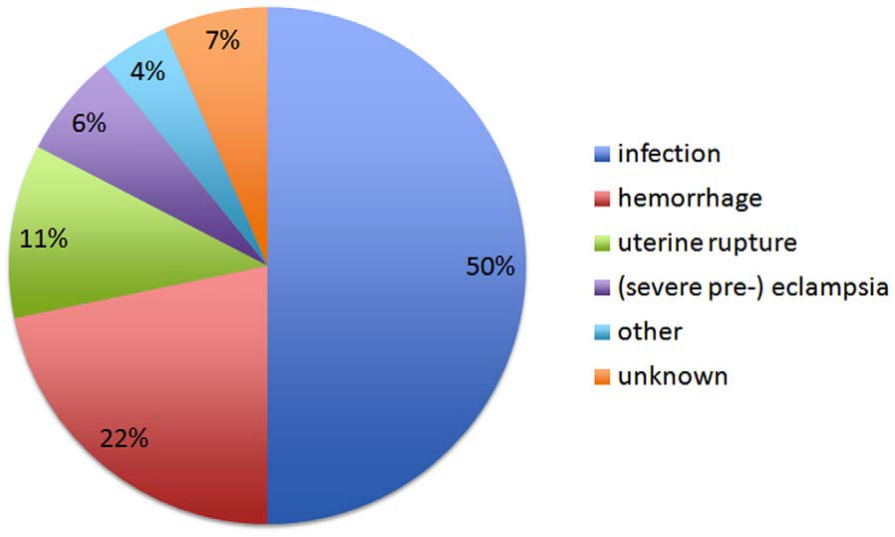
Maternal mortality and associated causes; n = 46.

Of the 46 women who died, 23 (50%) died of infection or severe complication of HIV-infection, ten (22%) of major obstetric hemorrhage, five (11%) of uterine rupture and three (7%) of eclampsia. Two died of other causes (sudden cardiac arrest during caesarean section and hepatocellular carcinoma) and in three cases the cause of death remained unclear (Figure 2). Of all 46 women who died, 19 (41%) were HIV-positive. Of the 23 women who died from infectious causes, eight (35%) had obstetric infections of whom four were HIV-positive, two HIV-negative and two had an unknown HIV-status. Thirteen (57%) women died from non-obstetric infections (of whom nine women were HIV-positive, three HIV-negative and one had an unknown HIV-status) and two from HIV-related Kaposi 's sarcoma.

Case fatality rates were 16% (23/142) for severe infection (9% for obstetric infection and 26% for non-obstetric infection), 10% for uterine rupture, 8% for major obstetric hemorrhage, 4% for (severe pre-) eclampsia. In HIV-positive women who sustained infection, the case fatality rates were 10% for obstetric infection (4/40) and 33% for non-obstetric infection (9/27).

In total, 45 cases were audited in plenary with hospital staff: 24 cases of maternal mortality and 21 cases of SAMM. Practically all hospital clinicians (medical doctors and clinical officers) and nurses had themselves been involved in the management of at least one of the audited cases. In 30 cases (67%) more than half of the auditors assessed care to have been below acceptable standards. A selection of substandard care factors identified at health facility level is shown in table 1; a selection of recommendations put forward by staff in table 2.

**Table 1:** Selection of substandard care factors identified.

1	Poor monitoring of labor by nurses/midwives at all levels with inadequate use of the labor graph.
2	Lack of clear and up-to-date protocols in health facilities.
3	Poor following of available protocols for obstetric emergencies by nurses/midwives and clinicians in health center and hospital (e.g. postpartum hemorrhage, shock, eclampsia).
4	Poor referral practices in health centers with delay in starting correct treatment, delay in obtaining intravenous access and fluid management, delay in calling for ambulance
5	Poorly functioning transport system with inefficient use of ambulances.
6	Delay at hospital level in assessing admitted emergency cases by nurses and clinicians and late arrival of hospital theatre staff for emergency procedures.
7	Poor postpartum and postoperative monitoring.

**Table 2:** Selection of recommendations by staff.

Ad 1	Refresher training on use of labor graph and emergency obstetric care, and increased supervision of labor and maternity wards by senior clinicians with daily or twice daily postpartum visits to critical patients by senior clinician.
Ad 2	Protocols on all relevant conditions updated and clearly visible on walls of all labor wards in the district.
Ad 3	Regular bed-side and classroom teaching to clinical officers and nurses/midwives on relevant subjects including emergency care by medical doctor; daily presenting of obstetric cases during morning handovers by clinician on night duty; follow-up charts for monitoring and treatment of eclampsia and severe pre-eclampsia.
Ad 4	Visits to health centers by seniors providing feedback on referral practices.
Ad 5	Increased supervision of district health management on utilization of ambulances.
Ad 6	Increased supervision by senior clinicians on standards of practice of all staff including theatre staff, especially during night- and weekend-call duties.
Ad 7	Daily or twice daily postpartum visits to critical patients and enhanced supervision of postnatal ward by medical doctor or senior clinician; implementation of theatre report form.
Ad all	Involving partners from non-governmental organizations in maternal and newborn care.

The following actions were taken based on these recommendations: (1) formal classroom refresher training on labor graph use, emergency obstetric care and labor monitoring was given twice during the study period with assistance from Médecins Sans Frontières Belgium, Malawi mission, Thyolo project (MSF); a selected group of health workers attended a drill course in advanced life-support in obstetrics (ALSO); ward supervision by senior clinicians was scaled up; (2) updated and appropriate district protocols were posted in each health facility (where unavailable or outdated WHO-protocols were used), presence of these protocols was checked during quarterly supervision rounds with additional logistics and human resources provided by MSF; (3) regular on-job coaching was provided including bedside teachings by medical doctors, quality and attendance of morning handovers was enhanced, follow-up charts for hypertensive disorders were implemented (courtesy Dr. T. Meguid, [29]) and their use was monitored by ward supervisors; (4) if audit resulted in recommendations for the primary care level, the relevant health centers were visited and given direct personal feedback by senior health workers who were usually members of the district health management team; health centers were also assessed during the regular supervision rounds for appropriate record keeping and the availability of material resources and other requirements for maternal care; (5) ambulance use became more stringently monitored, with help from MSF, but increasing the efficacy of the transport system remained a challenge due to regular breakdown of vehicles and a lack of resources for maintenance and repair; (6) standards-of-practice were more closely monitored by senior clinicians and district health management and oral and written warnings were given in case of misconduct; (7) visits to critical patients were performed at least daily, and usually twice daily, by senior clinicians, including on weekend days.

**Figure 3:**
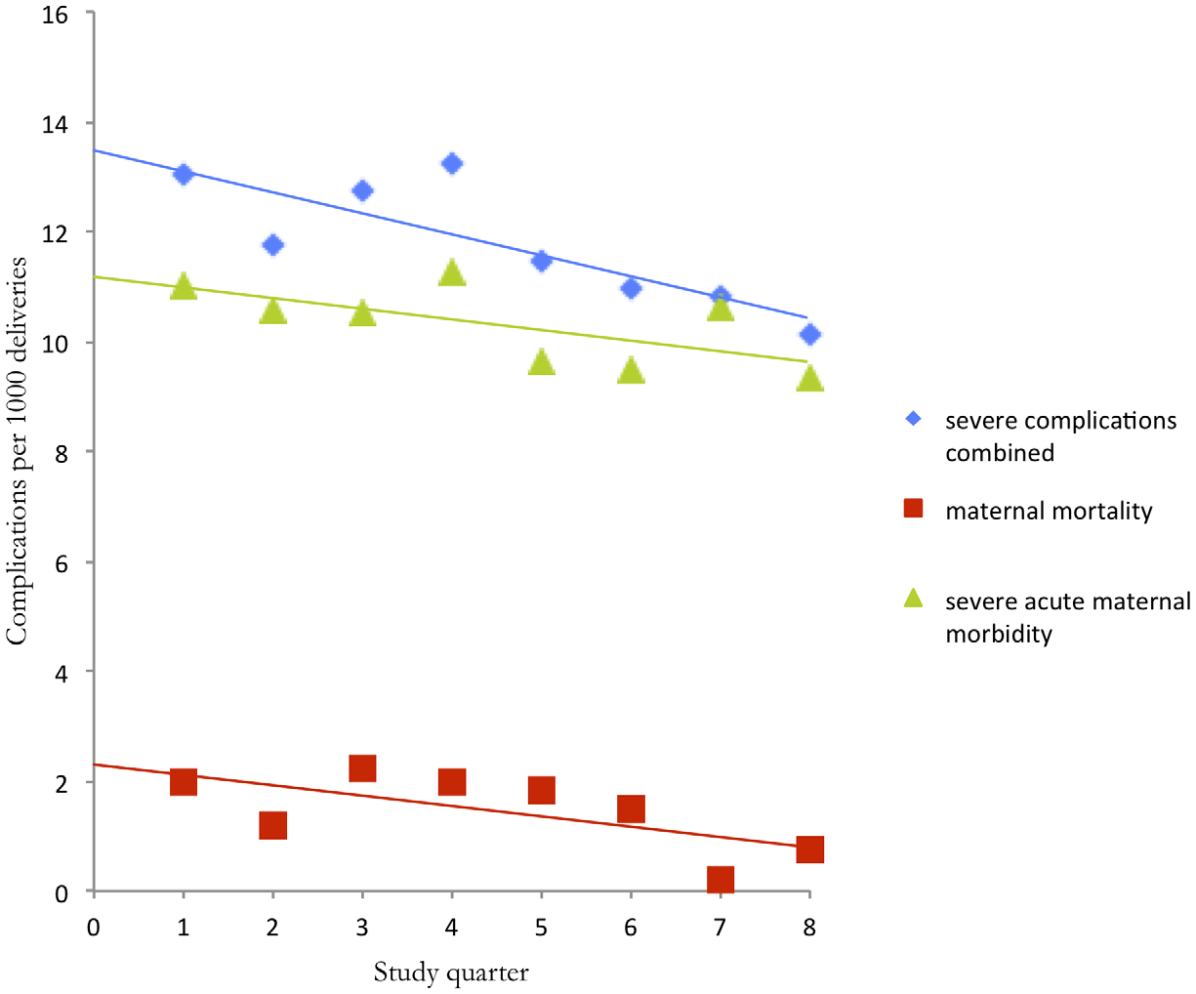
Women with severe maternal complications.

Table 3 shows the number of women who died or sustained SAMM per quarter throughout the study period. A linear regression identified a reduction of 3.1 inclusions for severe maternal complications (SAMM and mortality combined) per 1000 deliveries (regression coefficient −0.382; p = 0.01) from 13.5 to 10.4 complications per 1000 deliveries over the two-year period in which 33 254 facility-deliveries occurred in the district. This means that in (0.5 * 3.1 * (33 254/1000) = ) 52 women severe maternal complications were averted. The number of HIV-positive women did not change significantly (regression coefficient −0.196; p = 0.254). Linear regression analyses for facility-based maternal deaths and the number of women with SAMM showed downward trends for both (regression coefficients −0.189 per 1000 deliveries per quarter (p = 0.07) and −0.194 per 1000 deliveries per quarter (p = 0.08) respectively) (Figure 3).

**Table 3:** Number of study inclusions per quarter.

Quarter	facility deliveries	women included		maternal deaths		women with SAMM		HIV-positive women	
		N	per 1000	N	per 1000	n	per 1000	n	per 1000
1	2995	39	13.02	6	2.00	33	11.02	9	3.01
2	3404	40	11.75	4	1.18	36	10.58	16	4.70
3	3616	46	12.72	8	2.21	38	10.51	15	4.15
4	4078	54	13.24	8	1.96	46	11.28	23	5.64
5	4366	50	11.45	8	1.85	42	9.62	14	3.21
6	4746	52	10.96	7	1.47	45	9.48	15	3.16
7	4808	52	10.82	1	0.21	51	10.61	13	2.70
8	5241	53	10.11	4	0.76	49	9.35	15	2.86
Total	33 254	386	11.61	46	1.38	340	10.22	120	3.61
Regression coefficient, p-value			−0.382, 0.01		−0.189, 0.07		−0.194, 0.08		−0.196, 0.254

The numbers of diagnoses made in each diagnostic category per quarter, combining SAMM and mortality, are shown in table 4. There were significant reductions in hemorrhages from 5.9 to 2.6 per 1000 deliveries (regression coefficient −0.411; p = 0.006) and uterine ruptures from 3.5 to 0.2 per 1000 deliveries (regression coefficient −0.417; p = 0.036). No significant trends were found for (pre-) eclampsia (regression coefficient 0.108; p = 0.330) or peripartum infections (regression coefficient −0.078; p = 0.62).

**Table 4:** Number of diagnoses per category per quarter (SAMM and maternal mortality combined).

Quarter	facility deliveries	Infections		major hemorrhage		(severe pre-) eclampsia		uterine rupture	
		n	per 1000	n	per 1000	n	per 1000	n	per 1000
1	2995	10	3.34	17	5.68	6	2.00	14	4.67
2	3404	17	4.99	15	4.41	5	1.47	7	2.06
3	3616	18	4.98	16	4.42	9	2.49	6	1.66
4	4078	18	4.41	21	5.15	11	2.70	5	1.23
5	4366	18	4.12	17	3.89	13	2.98	3	0.69
6	4746	26	5.48	18	3.79	6	1.26	3	0.63
7	4808	21	4.37	10	2.08	12	2.50	6	1.25
8	5241	14	2.67	15	2.86	16	3.05	4	0.76
Total	33 254	142	4.27	129	3.88	78	2.35	48	1.44
Regression coefficient, p-value			−0.078, 0.62		−0.411, 0.006		0.108, 0.33		−0.417, 0.036

Uterine ruptures at district hospital level further decreased to 3.7 per 1000 hospital deliveries (excluding deliveries at peripheral primary care centers) in the second year of study, from 6.1 per 1000 after audit in the first year and an initial pre-audit incidence of 19.2 per 1000 hospital deliveries [9].

## Discussion

To our knowledge this is one of the first studies quantifying improvements in maternal outcome during a prolonged period of systematic audit on a district-wide scale in sub-Saharan Africa [7], [30], [31]. Although the study design does not allow for the establishment of a definite causal relationship between the observed reduction in maternal complications and obstetric audit, our findings add importantly to the limited body-of-evidence indicating that audit and feedback may reduce severe maternal complications at district level. In light of the absence of randomized trials and given the challenges to document and report complications in under-resourced settings, we advocate the continued implementation of obstetric audit based on these findings [4], [32]. The morbidity/mortality-ratio of 7.4 is similar to several other international studies, although this ratio is highly variable between different studies as a result of the absence of international SAMM criteria [17].

A number of possible study limitations merit discussion. First, our study setting is a real-time district health care system rather than a controlled study environment. Therefore, our findings do not allow for the identification of a causal relationship between audit and maternal outcome. However, since audit and feedback were the most significant injection into the quality of obstetric care in the district, the progressive and significant decline in severe maternal complications is likely to be a result of their implementation. Other factors are unlikely to have had significant impact. The socioeconomic and demographic characteristics of the district population were unlikely to have changed much over these two years.

Second, it is possible that the eagerness among staff to identify and include cases over time decreased. This is unlikely, however, due to the fact that the health worker responsible for study inclusions rotated on a two- to three-monthly basis. Moreover, these data collection staff were unaware of the objective to perform a trend analysis and were always supervised by the same team of dedicated senior clinicians.

Nevertheless, it is important to note that our SAMM and mortality figures are minimum figures. Like in any other operational research study underreporting may have occurred [33], particularly as some women may have had complications in peripheral facilities and failed to be referred or adequately recorded. Due to human resource and logistic constraints it was not possible to perform a detailed assessment of all delivery records in all peripheral facilities to acquire a better idea of underreporting at primary care level. However, from the sample assessed it appeared that underreporting was low. Also, it is unlikely that the frequency of underreporting would have changed much throughout the study period. In a similar nation-wide study from The Netherlands, underreporting was 2 and 3% for uterine rupture and eclampsia respectively, and 29% for major hemorrhage [26]. In our single-location study setting, the underreporting of hemorrhage is likely to be lower. We think it is unlikely that more than one or two cases of SAMM would have been underreported every quarter, and underreporting of mortality is even more unlikely. Such low rates of underreporting would not alter our conclusions.

The idea that audit and feedback were the main driver behind the decline in severe maternal complications is enhanced by the finding that the major reductions were found in the incidence figures for major obstetric hemorrhage and uterine rupture. These are the complications on which improved intrapartum care will have the most direct impact. Since audit and feedback focused primarily on improved intrapartum care and health worker performance during obstetric emergencies, it is not surprising that the declines in major hemorrhage and uterine rupture were significant and contrasted with the trends for eclampsia and peripartum infection.

Prevention of eclampsia will require increased uptake of appropriate antenatal care. Both an increased uptake and an improved quality of antenatal care are notoriously difficult to achieve. Lack of health worker time, irregular antenatal attendance and faulty blood pressure equipment prohibited significant improvements in antenatal blood pressure monitoring in Thyolo.

Since the HIV-prevalence among women with peripartum infections was high (almost 50%), it is likely that the incidence of these infections will reduce as uptake of HIV-treatment increases. In Thyolo, antiretroviral therapy (ART) was scaled up since 2003 with a positive impact on the survival of HIV-positive (pregnant) women as well as on the availability of health workers [34], [35]. The scaling up of ART was completed by August 2007 (before the beginning of the study period) and sustained thereafter without a further increase in ART-uptake [34]. Based on this information, it is likely that the most significant reduction in peripartum infections due to increased uptake of HIV-treatment took place already before the onset of this study (this is confirmed by anecdotal evidence from district health workers). The impact of HIV-treatment on a further reduction of peripartum infections is likely to become evident over a longer study period with a larger study sample.

In conclusion, audit of SAMM can be a useful tool for district health workers and managers, with the capacity to tangibly improve maternal outcome. By addressing health professionals' behavior audit has the capacity to avoid phase-III delay. If health managers and those responsible for transport are part of audit, phase-II delay may also be reduced. And eventually, if improvements in obstetric care are noticed throughout the community, audit may even positively influence care-seeking behavior and lead to a decrease of phase-I delay [2]. However, the impact of audit on pregnancy outcome as well as on staff motivation and performance warrants additional quantitative as well as qualitative research. Further scrutiny of the role of infectious causes including HIV in maternal health is also required.

## Supporting Information

Box S1 **Rest group inclusions.**
(DOCX)
