# Supplementary material for: Reduction of Severe Acute Maternal Morbidity and Maternal Mortality in Thyolo District, Malawi: The Impact of Obstetric Audit
Source: PLoS One. 2011 Jun 3;6(6):e20776. doi: 10.1371/journal.pone.0020776 (PMC3109003; doi:10.1371/journal.pone.0020776)
Supplement: Box S1 — Rest group inclusions. (DOCX) [file pone.0020776.s001.docx]

Box 1. Rest group inclusions.

| The 19 inclusions in the rest group were: four postpartum psychoses, three cases of severe anemia of unknown cause, two ectopic pregnancies and a post-caesarean bleed which did not meet the criteria for major hemorrhage, an epileptic convulsion, a depression with suicide attempt, a stroke due to hypertension, a case of liver failure with unknown cause, a case of obstructed labor with bladder neck necrosis resulting in vesicovaginal fistula, a cardiac failure of unknown origin, a post-caesarean burst abdomen without visible infection, a uterine perforation due to unsafe abortion and a case of lactic acidosis as a side effect of antiretroviral therapy. |
| --- |
